# Prognostic significance of systemic immunoinflammatory biomarkers in patients with clear cell renal cell carcinoma: a retrospective multicenter analysis

**DOI:** 10.3389/fimmu.2025.1575497

**Published:** 2025-08-13

**Authors:** Danlei Chen, Zhuorui Zhang, Longsheng Dong, Zhiyuan Feng, Junfeng Yang

**Affiliations:** ^1^ Department of Urology, The First People’s Hospital of Yunnan Province, Kunming, China; ^2^ Department of Urology, The Affiliated Hospital of Kunming University of Science and Technology, Kunming, China

**Keywords:** ccRCC, ISUP, NLR, PLR, LMR, SII, SIRI

## Abstract

**Aims:**

This study aims to investigate the associations between blood laboratory findings, systemic immune-inflammatory factors, ISUP grade, and prognostic outcomes in patients diagnosed with clear cell renal cell carcinoma (ccRCC).

**Methods:**

Data were collected from patients diagnosed with ccRCC at the First People’s Hospital of Yunnan Province and Xiangya Hospital of Central South University between 2010 and 2022, encompassing pertinent pathological and clinical information. We employed ordered logistic regression analysis to establish the ISUP grading model and conducted association analyses to explore the relationship between ISUP classification and the 2-year survival rate.

**Results:**

A total of 1,635 patients were included in the study. Our findings indicate that ISUP grading is significantly correlated with serum albumin levels, blood chloride concentrations, the platelet-to-lymphocyte ratio (PLR), and the systemic inflammation response index (SIRI). The preoperative 2-year survival model demonstrated associations with platelet counts (PLT), albumin levels, blood chloride concentrations, and PLR, AUC (0.776 95%IC 0.710, 0.843). Furthermore, the postoperative 2-year survival model exhibited robust correlations with PLT, albumin levels, blood chloride concentrations, PLR, and ISUP grade, AUC (0.877, 95%IC 0.825, 0.917).

**Conclusion:**

This study demonstrated the relationship between systemic immune markers, ISUP classification, and prognosis. It established preoperative models for predicting ISUP classification, as well as models for predicting 2-year survival rates both preoperatively and postoperatively.

## 1 Introduction

Renal cell carcinoma (RCC) accounts for 2.2% of all cancer cases and 1.8% of global cancer incidence, with an increasing trend in recent years (1). RCC ranks among the ten most prevalent cancers in men and is the fourteenth most common cancer in women (2). The primary subtypes of renal cell carcinoma (RCC) include clear cell renal cell carcinoma (ccRCC), papillary renal cell carcinoma (pRCC), and chromophobe renal cell carcinoma (chRCC), with ccRCC being the most prevalent, accounting for approximately 90% of all cases (3). ccRCC is one of the most common tumors in the urinary system,characterized by its insidious onset and indolent progression. In the early stages, most patients exhibit no obvious symptoms and are often diagnosed incidentally during routine checkups (4).

To date, several clinical-pathological prognostic models, such as the TNM staging system (5), Fuhrman grading system (6), Mayo Clinic staging, size, grade, and necrosis (SSIGN) scoring (7) are commonly employed to predict the prognosis of postoperated renal cell carcinoma (RCC) patients. Despite their widespread application, accurate prediction of individual tumor biology and prognosis remains a challenging task. In recent years, systemic immune-inflammatory markers have gained attention as valuable tools for evaluating tumor grade and prognosis in solid tumors (8). One notable marker, the Neutrophil-to-lymphocyte ratio (NLR) reflects the ratio of neutrophils and lymphocytes in the blood, and therefore, it has been considered as a potential circulating biomarker of cancer-related host inflammation and has emerged as a significant indicator of cancer progression in recent years (9). NLR has been associated with poor prognosis in a variety of tumor types, including mesothelioma, bladder cancer, renal cell carcinoma, colorectal cancer, ovarian cancer, and gastric cancer (10). Similarly, the platelet-to-lymphocyte ratio (PLR), which has been investigated in various conditions, including solid tumors, systemic lupus erythematosus, coronary artery disease, retinal artery occlusion, chronic kidney disease, and stable chronic obstructive pulmonary disease, serves as a valuable tool in assessing cancer prognosis (11).

Moreover, the elevated lymphocyte-to-monocyte ratio (LMR) has also been explored as a potential predictor of poor cancer prognosis; however, the results across studies remain inconclusive due to variations in methodology and sample size (12).

The Systemic Immune-Inflammation Index (SII) is a novel biomarker for malignant tumors and inflammatory diseases (13). Together with the Systemic Inflammation Response Index (SIRI), they are both comprehensive and innovative inflammation biomarkers based on immune cell subpopulations and platelet counts (13). These indicators have been widely utilized to evaluate the relationship between chronic inflammatory state and various human diseases,including cancer, metabolic disorders, and inflammatory conditions (14). Researchers have conducted extensive studies on the occurrence, development and prognosis of renal cancer, including investigations into mTOR signaling, mitochondrial DNA variants, and pan-RCC molecular features using machine learning models (15). Despite the increasing interest in systemic immune-inflammatory markers, much of the current research is based on public database analyses, with limited practical application examples.

The World Health Organization (WHO)/International Society of Urological Pathology (ISUP) grading system is a critical prognostic factor for ccRCC (16). Current research mainly focuses on predicting the ISUP grade of ccRCC patients prior to treatment by employing machine learning techniques based on CT or MRI imaging (17–19). However, the relationship between systemic immune-inflammatory markers and the ISUP grading system remains unexplored.

This study explored the correlation between pre-treatment tests, systemic immune-inflammatory markers, ISUP grade, and the two-year survival rate of ccRCC. Our goal is to develop a predictive model by integrating these biomarkers with clinical data, which can facilitate personalized treatment planning and enhance clinical decision-making.

## 2 Methods

### 2.1 Patients

This study was approved by the Ethics Committee of the First People’s Hospital of Yunnan Province. Due to the retrospective study, informed consent was not required for this study. The study follows the principles outlined in the Helsinki Declaration (2013 revision). A comprehensive search of pathology databases identified 1589 patients with ccRCC who underwent kidney tumor resection at the facility between January 2010 and June 2022. Patients who were followed for less than two years, as well as those with incomplete laboratory data, were excluded. Ultimately, 1527 patients were included in the study of the correlation between biochemical and immunoinflammatory indicators and ISUP grading. Additionally, 1,154 patients were included in the study of biochemical indicators and immunoinflammatory indicators and 2-year overall survival rate (OS).

### 2.2 Pathological review

Two experienced pathologists (Y Wang and YN Li, with 20 and 18 years of experience in uropathology) retrospectively analyzed all pathological data of 1527 patients to to confirm both the diagnosis of ccRCC and the ISUP grade. In the event of a discordance among different histopathological slides, the two pathologists jointly reevaluated the sections until a consensus is reached. The reviewers were unaware of the clinical diagnostic information.

### 2.3 Data collection

We extract demographic and clinical data from medical records, including gender, age of onset, BMI before surgery, and a wide range of laboratory parameters, including blood cell counts, biochemical markers, and blood trace elements. Also, we gathered postoperative follow-up data and time of death from the records.

### 2.4 Statistical analysis

S Statistical analyses were performed using IBM SPSS Statistics version 27.0 (IBM SPSS Inc., Chicago, IL, USA) and R software version 4.0.1 (http://www.r-project.org). Continuous variables with a normal distribution were expressed as mean (standard deviation), and Student’s t-test was used for analysis. Non-normally distributed variables were expressed as median (interquartile range), and the Mann-Whitney U test was applied. Categorical variables were compared using Pearson’s chi-square test or Fisher’s exact test. The critical values of inflammatory response factors were determined by the area under the receiver operating characteristic (ROC) curve (AUC) based on ISUP grading, and the factors were dichotomized according to the critical values. Ordinal univariate and multivariate logistic regression analyses were conducted to identify factors associated with ccRCC and ISUP grading. Variables with P < 0.05 in the univariate analysis were considered candidate variables for the multivariate analysis and retained in the model. Predictive models for 2-year overall survival in ccRCC were developed using the identified ISUP model and evaluated for discrimination and calibration. The AUC was used to assess discriminatory ability, and calibration was tested by comparing observed and predicted probabilities. A two-sided P value < 0.05 was considered statistically significant.

## 3 Result

### 3.1 Baseline characteristics

A total of 1,635 evaluable patients were included in this study, of which 1,126 (68.9%) were male. Among the participants, 1,492 patients underwent laparoscopic minimally invasive surgery, constituting 91.5% of the cohort. Additionally, 54.0% of patients were classified as ISUP grade 2. During the follow-up period, 37 patients (2.3%) experienced recurrence, while 113 patients (6.9%) developed metastases. In total, 140 patients (8.6%) developed either local recurrence or distant metastases (**Table 1**).

**Table 1:** Basic features of ccRCC.

Character	n (%)
Gender	
Male	1126 (68.9%)
Female	508 (31.1%)
Location	
Left	804 (49.4%)
Right	824 (50.6%)
Surgical method selection	
Laparoscopic surgery	1492 (91.5%)
Traditional surgery	139 (8.5%)
Radical surgery	959 (58.9%)
Partial resection	669 (41.1%)
ISUP	
1	372 (23.5%)
2	855 (54.0%)
3	290 (18.3%)
4	66 (4.2%)
Cytopathologic change	
Necrosis	266 (16.3%)
Giant cell change	20 (1.2%)
Cystic changes	331 (20.2%)
Intracellular hemorrhage	594 (36.3%)
Vascular tumor thrombus	40 (2.4%)
Recurrence	37 (2.3%)
Metastases	113 (6.9%)
Recurrence or Metastasis	140 (8.6%)

In patients with ccRCC, the proportions of abnormal red blood cells (RBC) count were found to be 27.7%, abnormal hemoglobin (HGB) at 31.7%, and eosinophils (EO) at 21.0%, indicating a relatively high level (**Table 2**). Regarding common biochemical tests, the proportions of abnormal total protein (TP) at 25.9%, albumin (ALB) at 29.5%, globulin (GLB) at 21.3%, and uric acid (UA) at 39.3%, all of which were notably elevated (**Table 3**). Furthermore, the proportion of patients with hyperglycemia was 20.8%, and the proportion of patients with hypokalemia was 9.6% (**Table 4**).

**Table 2:** Basic characteristics of blood routine in ccRCC.

Character	n (%)
White blood cells (WBC)	
High	196 (12.4%)
Low	29 (1.8%)
Red blood cells (RBC)	
High	57 (3.6%)
Low	381 (24.1%)
Hemoglobin (HGB)	
High	8 (0.5%)
Low	492 (31.2%)
Platelet (PLT)	
High	160 (10.1%)
Low	34 (2.2%)
Neutrophils (NEU)	
High	173 (11.0%)
Low	52 (3.3%)
Lymphocytes (LY)	
High	110 (7.0%)
Low	0
Eosinophils (EO)	
High	37 (2.3%)
Low	295 (18.7%)
Basophilic granulocyte (BASO)	13 (0.8%)
Monocytes (MONO)	
High	101 (6.4%)
Low	34 (2.2%)

**Table 3:** Basic characteristics of biochemical routine of ccRCC.

Character	n (%)
Total protein (TP)	
High	38 (2.4%)
Low	368 (23.5%)
Albumin (ALB)	
High	8 (0.5%)
Low	454 (29%)
Globulin (GLB)	
High	215 (13.7%)
Low	119 (7.6%)
Total bilirubin (TBIL)	
High	191 (12.2%)
Low	2 (0.1%)
Direct bilirubin (DBIL) (high)	208 (13.3%)
Blood Urea Nitrogen (BUN)	
High	195 (12.5%)
Low	59 (3.8%)
Creatinine (Crea)	
High	95 (6.1%)
Low	35 (2.2%)
Uric acid (UA)	
High	594 (38.0%)
Low	21 (1.3)

**Table 4:** Basic characteristics of blood glucose and biochemical routine in ccRCC.

Character	n (%)
Glucose (GLU)	
High	326 (20.8%)
Low	7 (0.4%)
K	
High	7 (0.4%)
Low	151 (9.6%)
Na	
High	5 (0.3%)
Low	54 (3.5%)
Cl	
High	38 (2.4%)
Low	85 (5.4%)
Ca	
High	27 (1.7%)
Low	27 (1.7%)
P	
High	17 (1.1%)
Low	45 (2.9%)
Mg	
High	13 (0.8%)
Low	15 (1.0%)

In this study, the median values of various systemic immune-inflammation markers were as follows: NLR 2.5298 (1.7778, 3.9110), PLR 132.9289 (97.5000, 180.0000), LMR 3.3333 (2.3333, 4.5000), SII 77.1556 (48.2222, 116.0857), and SIRI 1.1250 (0.7385, 2.0698) (**Table 5**).

**Table 5:** Basic characteristics of coagulation routine and systemic immunoinflammatory markers in ccRCC.

Character	n
Prothrombin time (PT) (%)	
High	42 (2.7%)
Low	13 (0.8%)
Activated partial thromboplastin time (APTT) (%)	
High	14 (0.9%)
Low	3 (0.2%)
NLR, median (IQR)	2.5298 (1.7778, 3.9110)
PLR, median (IQR)	132.9289 (97.5000, 180.0000)
LMR, median (IQR)	3.3333 (2.3333, 4.5000)
SII, median (IQR)	77.1556 (48.2222, 116.0857)
SIRI, median (IQR)	1.1250 (0.7385, 2.0698)

### 3.2 Systemic immunoinflammatory biomarkers & ISUP

A total of 1527 patients were enrolled in the final study. In patients with ccRCC, non-parametric analysis of ISUP grading with common blood tests revealed the following associations. ISUP grading was related to red blood cells, hemoglobin, platelets, neutrophils, lymphocytes, monocytes, total protein, albumin, total bilirubin, direct bilirubin, blood sodium, blood chloride, blood calcium, blood phosphorus, blood magnesium, prothrombin time, fibrinogen, D-dimer, NLR, PLR, LMR, SII, SIRI. Common blood tests were partitioned in normal/abnormal, and the maximum area under the ROC curve was calculated for inflammatory response markers according to ISUP classification. The threshold value was selected according to the maximum area for classification. The optimal cut-off values of all systemic immune-inflammation markers for all patients were determined based on the Youden index, which were as follows: NLR 3.05, PLR 138.86, LMR 8.17, SII 99.60, and SIRI 1.22. Based on these values, the patients were then categorized into low and high groups. After classification, the critical values of NLR, PLR, LMR, SII and SIRI were determined respectively. The Chi-square test shows that ISUP grading correlates with RBC, HGB, PLT, LY, ALB, blood chloride concentration, PT, fibrinogen, NLR, PLR, and SIRI (**Table 6**).

**Table 6:** Correlation of biochemical biomarkers and systemic immune inflammatory markers with ISUP grading.

n=1527	p (Mann-Whitney U Test)	p (Chi-square test)
WBC	0.036	0.712
RBC	<0.001	<0.001
HGB	<0.001	<0.001
PLT	<0.001	<0.001
NEU	0.03	0.125
LY	0.009	0.027
EO	0.839	
BASO	0.667	
MONO	0.004	0.181
TP	0.038	0.163
ALB	<0.001	<0.001
TBIL	<0.001	0.118
DBIL	<0.001	0.069
BUN	0.897	
Crea	0.444	
UA	0.753	
GLU	0.112	
K	0.224	
Na	0.011	0.603
Cl	0.008	0.034
Ca	0.013	0.359
P	0.016	0.130
Mg	0.026	0.758
PT	0.007	0.037
Fg	0.027	0.021
APTT	0.061	
D-Dimer	<0.001	0.251
NLR	<0.001	<0.001
PLR	<0.001	<0.001
LMR	<0.001	0.058
SII	0.047	0.157
SIRI	<0.001	<0.001

Correlation factors were included in ordered logistics regression analysis. Through ordered univariate logistics regression analysis, we found that ISUP classification correlated with red blood cells, hemoglobin, PLT, lymphocytes, albumin, blood chloride concentration, prothrombin time, fibrinogen, NLR, PLR and SIRI. Further, orderly multivariate logistics regression revealed that ISUP classification correlated with albumin, blood chloride ion concentration, PLR and SIRI (**Table 7**).

**Table 7:** Orderly logistics regression analysis of ISUP grading.

n=1527	Ordinal univariate logistics regression analysis				Ordinal multivariate logistics regression analysis			
	p	OR	95%IC		p	OR	95%IC	
RBC	<0.001	1.562	1.259	1.939				
HGB	<0.001	1.062	1.271	1.610				
PLT	<0.001	1.716	1.281	2.300				
LY	0.037	1.259	1.014	1.562				
ALB	<0.001	1.430	1.267	1.613	<0.001	1.283	1.133	1.452
Cl	0.009	1.340	1.070	1.605	0.039	1.241	1.011	1.523
PT	0.035	1.852	1.044	3.284				
Fg	0.041	1.362	1.013	1.831				
NLR	<0.001	1.269	1.134	1.420				
PLR	<0.001	1.362	1.220	1.520	0.002	1.208	1.073	1.359
SIRI	<0.001	1.381	1.236	1.542	<0.001	1.220	1.083	1.374

### 3.3 Systemic immunoinflammatory biomarkers & 2-year OS

A total of 1154 patients were enrolled in the final study. In patients with ccRCC, the 2-year overall survival (OS) was assessed using a Chi-square test in relation to common blood tests. The results showed that 2-year OS was correlated with red blood cell, hemoglobin, lymphocyte, basophil, monocyte, albumin, creatinine, blood sodium, blood chloride, blood calcium, blood magnesium, thrombin time, NLR, PLR, SIRI, and ISUP grades (**Table 8**).

**Table 8:** Correlation of biochemical biomarkers and systemic immune inflammatory biomarkers with 2-year OS.

n=1154	p
WBC	0.317
RBC	<0.001
HGB	<0.001
PLT	<0.001
NEU	0.835
LY	0.007
EO	0.122
BASO	0.013
MONO	0.018
TP	0.344
ALB	<0.001
	0.19
TBIL	0.366
DBIL	0.93
BUN	0.001
Crea	<0.001
UA	0.692
GLU	0.548
K	0.184
Na	0.004
Cl	<0.001
Ca	0.001
P	0.131
Mg	<0.001
PT	0.093
APTT	0.002
NLR	0.01
PLR	<0.001
LMR	0.227
SII	0.223
SIRI	<0.001
IUSP	<0.001

**Figure 1:**
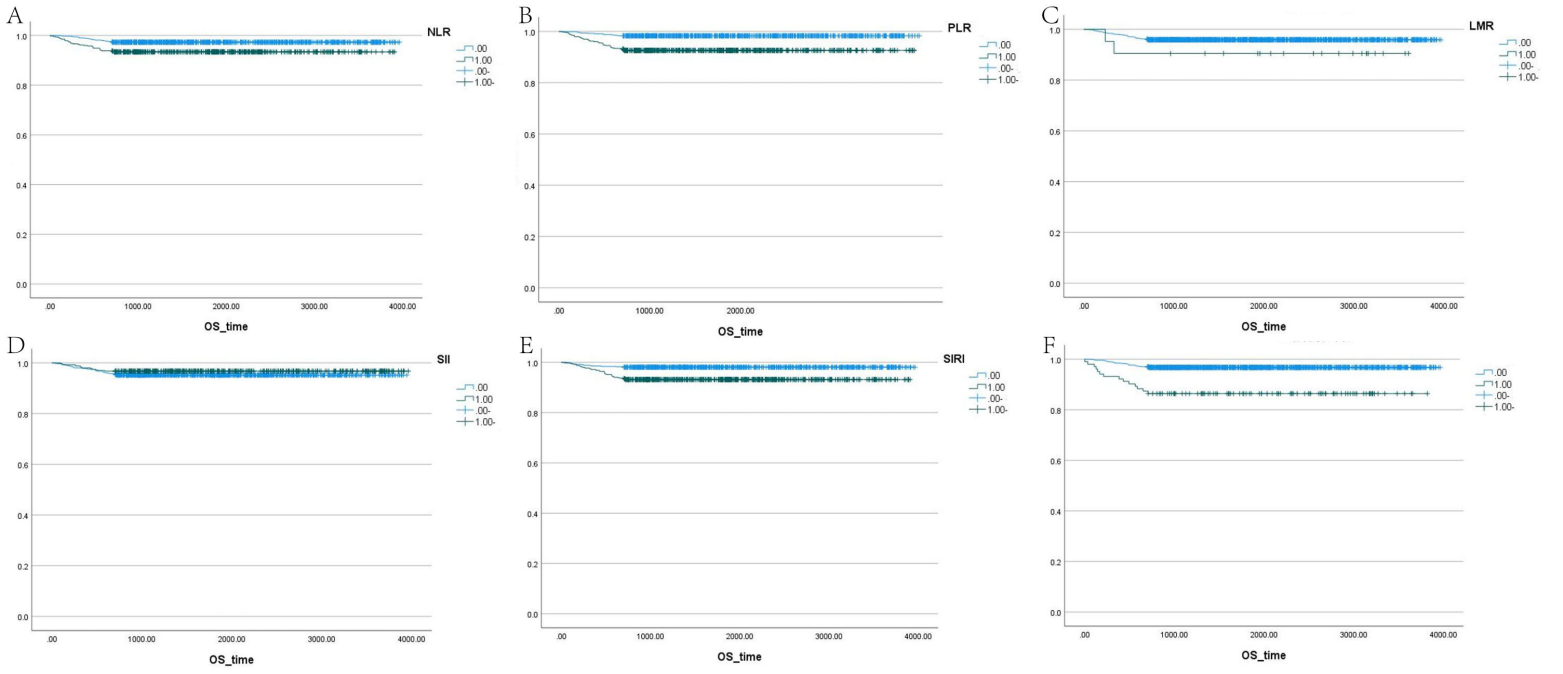
Survival analysis of LMR, SII, NLR, PLR, SIRI, and blood chloride. **(A–F)** Each panel represents the Kaplan-Meier survival curves for NLR, PLR, LMR, SII, SIRI, and blood chloride levels, respectively.

**Figure 2:**
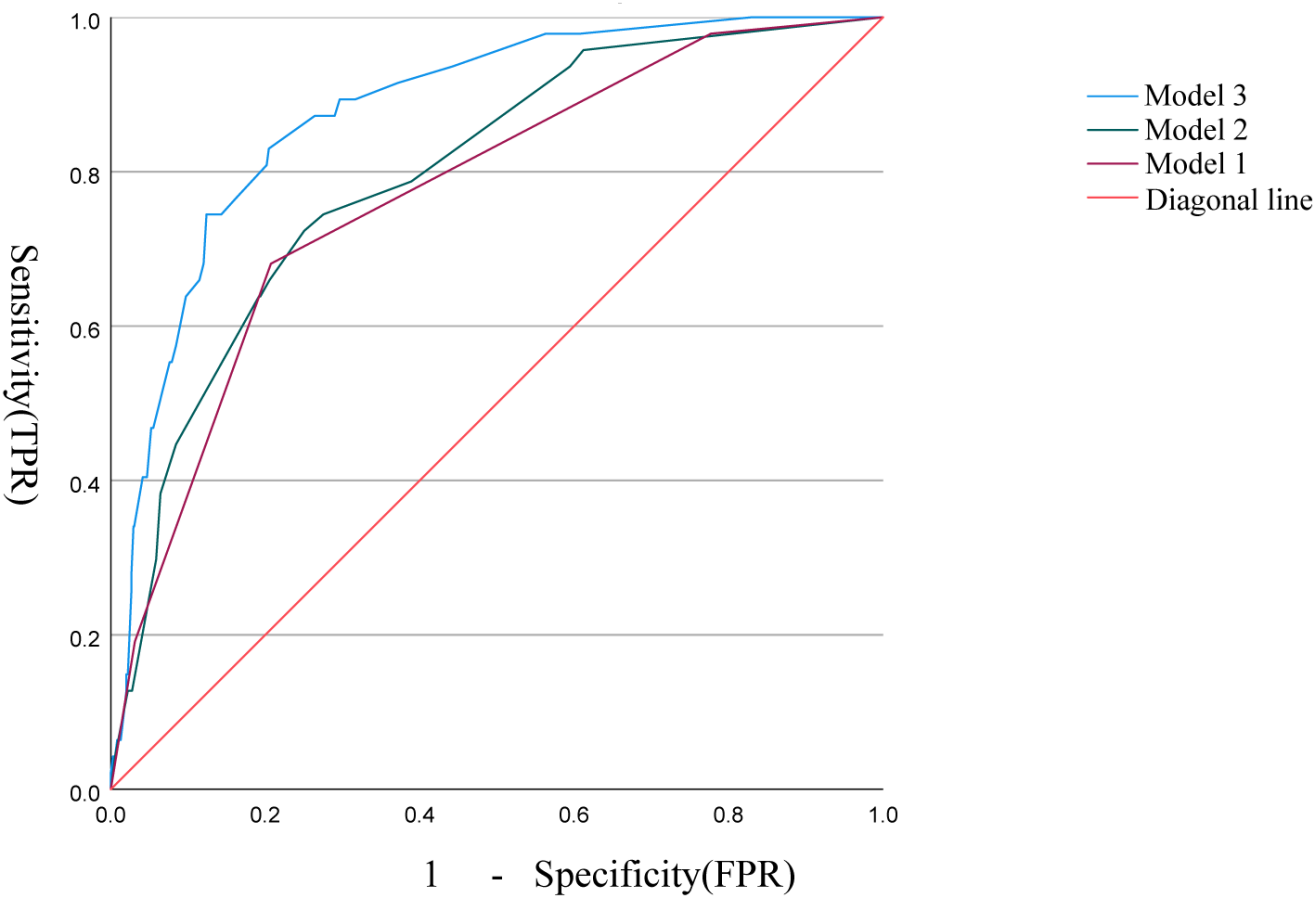
Comparison of sensitivity and specificity between the three models.

The correlation factors were included in the logistics regression analysis. The univariate logistics regression analysis showed that the 2-year OS was correlated with RBC, HGB, PLT, ALB, BUN, blood chloride, APTT, NLR, PLR, SIRI and ISUP grades (**Table 9**). Furthermore, a KM survival curve analysis was conducted for the relevant factors, and the results showed that the 2-year survival rate was correlated with NLR (p=0.001), PLR (p<0.001), SIRI (p<0.001), and blood chloride (p<0.001), but not significantly related to LMR (p=0.215) and SII (p=0.221) (**Figure 1**). Relevant factors were incorporated into the model to predict the 2-year OS. Model 1 used ISUP grading to predict 2-year OS. Model 2 utilized preoperative test data and systemic immunoinflammatory index to predict 2-year OS (**Table 10**). Model 3 was developed by combining the data from Model 1 and Model 2 (**Table 11**). The AUC of model 1 is 0.773, 95%IC (0.705, 0.841). The AUC of Model 2 is 0.796, 95%IC (0.733, 0.859). The AUC of Model 3 is 0.878, 95%IC (0.833, 0.923) (**Table 12**, **Figure 2**).

**Table 9:** Univariate logistic regression analysis of 2-year OS.

n=1154	p	OR	95%IC	
RBC	<0.001	3.305	1.862	5.865
HGB	<0.001	4.105	2.256	7.486
PLT	<0.001	3.804	2.062	7.018
ALB	<0.001	4.537	2.484	8.287
BUN	0.002	2.987	1.479	6.032
Cl	<0.001	4.714	2.439	9.113
PT	0.114	3.384	0.747	15.339
APTT	0.004	3.884	1.556	9.691
NLR	0.002	2.528	1.410	4.531
PLR	<0.001	4.548	2.305	8.973
LMR	0.242	2.428	0.549	10.729
SII	0.225	0.672	0.353	1.278
SIRI	<0.001	3.670	1.898	7.098
ISUP	<0.001	3.784	2.638	5.428

**Table 10:** Multivariate logistics regression analysis of 2-year OS for model 2.

Character	p	OR	95%IC	
PLT	0.029	2.123	1.081	4.170
ALB	<0.001	3.219	1.844	6.019
Cl	<0.001	3.745	1.866	7.478
PLR	0.002	2.985	1.651	6.064

**Table 11:** Multivariate logistics regression analysis of 2-year OS for model 3.

Character	p	OR	95%IC	
PLT	0.040	2.105	1.036	4.277
ALB	0.005	2.560	1.333	4.916
Cl	<0.001	4.116	1.959	8.650
PLR	0.012	2.527	1.224	5.219
ISUP	<0.001	3.180	2.152	4.699

**Table 12:** Comparison of predictive performance of three models.

Character	AUC	95%IC	
Model 1	0.773	0.705	0.841
Model 2	0.796	0.733	0.859
Model 3	0.878	0.833	0.923

## 4 Discussion

There is a complex interaction between systemic inflammatory response and tumor progression. It can enable tumor cells to evade immune surveillance, inhibit apoptosis, and enhance the possibility of genomic mutations, angiogenesis, invasion and metastasis in tumor patients (20–22). Neutrophils, lymphocytes and monocytes can affect tumor occurrence and progression by participating in the systemic inflammatory response (23).

In this study, serum albumin, blood chloride concentration, PLR and SIRI were identified as independent factors related to ISUP in patients with ccRCC. Although red blood cells, hemoglobin, platelets, lymphocytes, prothrombin time, fibrinogen and NLR were significantly associated with ISUP grade in univariate analysis, they were not retained as independent indicators in the multivariate model.

Furthermore, this study interestingly demonstrated that the 2-year overall survival rate was associated with PLT, albumin, blood chloride concentration, PLR grade and ISUP grade. The low levels of serum albumin, which is synthesized by the liver, not only represents a state of malnutrition but also indicates a persistent systemic inflammatory response (24). This study partially corroborates these findings.

However, LMR was not correlated with ISUP grade or 2-year survival rate in this study, which may be due to the selection of the critical value of LMR (25). The critical value of LMR was determined based on the maximum AUC after ISUP grading. Some studies suggest that an elevated NLR could be a negative prognostic indicator for metastatic ccRCC patients (26, 27). However, conflicting research findings exist (28). In this study, we found that NLR was associated with ISUP grade and 2-year survival rate but was not an independent factor.

Increasing evidence indicates that platelets may play a crucial role in inflammation, tumor occurrence and progression, potentially involving various growth factors such as platelet-derived growth factor and vascular endothelial growth factor (29, 30). It is speculated that platelets interact with tumor cells and release pro-angiogenic factors such as vascular endothelial growth factor, creating a favorable microenvironment for tumor growth by angiogenesis (31). The increase in inflammatory cytokine levels can also promote platelet production, and some evidence also suggests that platelets limit the ability of natural killer cells to clear tumor cells (32). Our findings further support the role of platelets in cancer progression, as platelet count correlates with ISUP grading and 2-year survival rates in patients. This hypothetical mechanism may help explain how elevated platelet counts are associated with poor prognosis in cancer patients.

Only one study has demonstrated the prognostic value of SII in patients with ccRCC combined with venous tumor thrombus (33). In this study, we failed to confirm the relationship between SII and ccRCC. Some studies have suggested that red blood cells can be used to evaluate the effectiveness of second-line nivolumab treatment in metastatic renal cell carcinoma (34). In this study, red blood cells and hemoglobin were factors affecting ISUP grade and survival rate but were not independent factors.

Lymphocytes play a significant role in cellular immunity and may promote the clearance of malignant cells (35). Although the research on the prognostic value of the platelet-to-lymphocyte ratio (PLR) in tumors has been widely pursued by researchers, studies on the pathological grading of tumors in relation to PLR are relatively scarce. Until now, only one study has investigated PLR in relation to prostate malignancies. In our study, PLR was found to be associated not only with the ISUP grade of ccRCC but also with the 2-year survival rate.This may provide a target for future research directions on systemic immune-inflammation markers.

Previous studies have shown that serum chloride ion concentration is associated with the prognosis of various cancers, including oral cancer (36), laryngeal cancer (37) and colon cancer (37). Additionally, there are studies demonstrating that the chloride ion concentration in cerebrospinal fluid can distinguish cerebral infarction from brain tumors (38). In renal cell carcinoma, some research has found that in patients with VHL gene mutations, the VHL gene can regulate tumor cell proliferation by controlling the Cl-/HCO3- exchange and Na+/H+ exchange activities in renal cancer cells (39). In this study, serum chloride ion concentration was found not only independently associated with ISUP grade, but also with 2-year survival rate. This finding may be a focus of future research on ccRCC. By overlooking the ion channels in previous studies and analyzing the correlation between serum chloride ion concentration and the prognosis of ccRCC from another perspective, we hypothesize that it is the invasion of renal tubules by renal tumors that affects the reabsorption of chloride ions. Our results also showed that patients with higher serum chloride ion concentrations often had higher ISUP grades and lower 2-year survival rates.

The ISUP grade is a critical determinant of prognosis of ccRCC. However, most existing studies on preoperative prediction of ISUP grade and prognosis primarily rely on imaging techniques, such as CT or MRI (17, 40, 41). The use of ROC-derived thresholds in these studies may lead to overfitting, reducing their external applicability. Additionally, the lack of molecular markers or imaging features known to influence prognosis limits model refinement. This study explored the relationship between routine blood laboratory tests and systemic immune-inflammation markers that affect prognosis and ISUP grade, and established a predictive model. Furthermore, based on the relationship between these factors and the ISUP grade, the study also established their connection with the 2-year survival rate and developed a predictive model.

This study offers a unique perspective in enhancing preoperative prediction models by incorporating the ISUP grade and 2-year survival rate. It also suggests that combining imaging data and blood laboratory tests of patients with ccRCC may provide a more accurate prognosis.

## 5 Conclusion

This study explored the relationship between hematological, laboratory biomarkers and systemic immune markers in patients with ccRCC and the ISUP grade and prognosis, it also established models for preoperative prediction of ISUP grade, preoperative prediction of 2-year survival rate and postoperative prediction of 2-year survival rate.

## Data Availability

The raw data supporting the conclusions of this article will be made available by the authors, without undue reservation.
